# The Effect of Everyday Stressors and the Perceived Stress Reactivity Scale on Variability in Sympathetic Arousal

**DOI:** 10.1093/geroni/igab046.2847

**Published:** 2021-12-17

**Authors:** MacKenzie Hughes, Christopher Hertzog, Shevaun Neupert, Scott Moffat

**Affiliations:** 1 Georgia Institute of Technology, Atlanta, Georgia, United States; 2 North Carolina State University, Raleigh, North Carolina, United States

## Abstract

This ecological momentary assessment study examined the effect of naturally occurring stressors and perceived stress reactivity on alpha-amylase, a proxy of sympathetic nervous system arousal. There are age-related changes in physiological systems sensitive to stress, so the sample included 174 adults ages 20-78 (M=48.65, SD=19.28). At the beginning of the study, participants completed the Perceived Stress Reactivity Scale (PSRS; Schulz et al., 2005). For 10 consecutive days, participants were prompted five times per day to report exposure to stressors. During the same 10-day period, participants provided seven saliva samples per day, assayed for alpha-amylase. Multilevel modeling was used to examine daily and momentary associations between stressors, the PSRS, and alpha-amylase activity. On a daily basis, stressors did not predict changes in the diurnal alpha-amylase pattern, but higher perceived stress reactivity predicted steeper diurnal slopes and lower total daily output. A significant cross-level interaction emerged showing people higher in perceived stress reactivity had steeper awakening responses on days they experienced more stressors than usual. On a momentary basis, alpha-amylase levels were higher on occasions when participants reported stressors. In addition, higher levels of perceived stress reactivity predicted lower overall alpha-amylase levels. Findings suggest that 1) stressors are associated with elevations in momentary but not daily aggregate levels of alpha-amylase, and 2) the PSRS has prospective validity as a predictor of stress-related fluctuations in diurnal alpha-amylase patterns. Age was not a significant moderator of the relationship between stressors and alpha-amylase, potentially suggesting the effect of stressors on alpha-amylase activity is age invariant.

